# Shift in trophic niches of soil microarthropods with conversion of tropical rainforest into plantations as indicated by stable isotopes (^15^N, ^13^C)

**DOI:** 10.1371/journal.pone.0224520

**Published:** 2019-10-25

**Authors:** Alena Krause, Dorothee Sandmann, Sarah L. Bluhm, Sergey Ermilov, Rahayu Widyastuti, Noor Farikhah Haneda, Stefan Scheu, Mark Maraun

**Affiliations:** 1 University of Göttingen, J.F. Blumenbach Institute of Zoology and Anthropology, Göttingen, Germany; 2 Tyumen State University, Tyumen, Russia; 3 Bogor Agricultural University-IPB, Department of Soil Sciences and Land Resources, Bogor, Indonesia; 4 Bogor Agricultural University-IPB, Department of Silviculture; Faculty of Forestry, Bogor, Indonesia; Universita del Salento, ITALY

## Abstract

Land-use change is threatening biodiversity worldwide, affecting above and below ground animal communities by altering their trophic niches. However, shifts in trophic niches with changes in land use are little studied and this applies in particular to belowground animals. Oribatid mites are among the most abundant soil animals, involved in decomposition processes and nutrient cycling. We analyzed shifts in trophic niches of six soil-living oribatid mite species with the conversion of lowland secondary rainforest into plantation systems of different land-use intensity (jungle rubber, rubber and oil palm monoculture plantation) in two regions of southwest Sumatra, Indonesia. We measured stable isotope ratios (^13^C/^12^C and ^15^N/^14^N) of single oribatid mite individuals and calculated shifts in stable isotope niches with changes in land use. Significant changes in stable isotope ratios in three of the six studied oribatid mite species indicated that these species shift their trophic niches with changes in land use. The trophic shift was either due to changes in trophic level (δ^15^N values), to changes in the use of basal resources (δ^13^C values) or to changes in both. The trophic shift generally was most pronounced between more natural systems (rainforest and jungle rubber) on one side and monoculture plantations systems (rubber and oil palm plantations) on the other, reflecting that the shifts were related to land-use intensity. Although trophic niches of the other three studied species did not differ significantly between land-use systems they followed a similar trend. Overall, the results suggest that colonization of very different ecosystems such as rainforest and intensively managed monoculture plantations by oribatid mite species likely is related to their ability to shift their trophic niches, i.e. to trophic plasticity.

## Introduction

Due to the worldwide growing human population and the associated rising need for food, fuel and fiber, transformation and degradation of landscapes rapidly increased over the last decades [1–4]. This is especially true for tropical regions where rainforest is rapidly and continuously transformed into different land-use systems, such as oil palm and rubber plantations [5–7]. Within the humid tropics Southeast Asia is one of the hotspots of recent deforestation with the highest loss of primary rainforest occurring in Sumatra (Indonesia) on average 0.40 Mha per year between 2009 and 2011 [8–10]. Notably, these hotspots of deforestation are located in regions with the highest biodiversity and highest level of endemism worldwide [6,11,12]. It has been shown that land-use intensification in the tropics affects diversity and biomass of soil animals including centipedes, earthworms and oribatid mites [5,13–17], which in turn may affect decomposition and nutrient cycling provided by these organisms. Oribatid mites are among of the most abundant soil arthropods worldwide and involved in decomposition processes and nutrient cycling [18,19]. There are more than 11,000 described species [20] with the true number of species likely exceeding 50,000 [21]. Oribatid mites can reach densities of up to 200,000 ind./m^2^ in forest soils of temperate regions whereas in tropical regions densities typically are in the range of 30,000–40,000 ind./m^2^ [18,22,23]. Oribatid mites are trophically diverse and stable isotope analyses suggest that they span over about four trophic levels including lichen feeders, fungal feeders, primary and secondary decomposers as well as predators/scavengers [24–26].

Trophic position and trophic interactions characterize species and their role in ecosystem functioning and services. For many ecosystem functions, such as decomposition, nutrient cycling, carbon sequestration, primary production and crop yield, the soil decomposer system is essential [27,28]. The trophic structure of animal communities can be evaluated by analyzing natural variations in ^15^N/^14^N and ^13^C/^12^C ratios [29–31]. Animal tissue typically is enriched in ^15^N as compared to their food resource by about 3 and for ^13^C by about 1 δ unit per trophic level, however, the enrichment may vary between trophic guilds and also between taxa [30,32,33]. Thereby, ^15^N values allow estimating trophic levels [34,35], whereas ^13^C is used to identify basal food resources since ^13^C values change little across trophic levels [29]. Stable isotopes have been used widely to analyze trophic niches of soil invertebrates [22,36–39] including earthworms [40], ants [41], springtails [42], gamasid mites [43] and oribatid mites [17,26,38]. However, until today stable isotopes rarely have been used to investigate how trophic niches of soil animal taxa are affected by changes in land-use [44,45].

Forest transformation and land-use intensification strongly affect animal and plant taxa, and the changes typically are associated by the loss of species [46–48]. An important mechanism to cope with environmental alterations such as land-use change is to respond in a plastic way by shifting trophic niches and adapt to the resources available locally. Trophic plasticity, therefore, may prevent extinction and thereby support biodiversity in converted ecosystems. Until today ecological plasticity mostly has been investigated in aquatic taxa, such as fish [49–51] and gastropods [52]. These studies, however, focused on changes in morphology and behavior due to changing environmental factors rather than on trophic plasticity. Few studies investigated trophic shifts in soil animals. Klarner et al. [13] showed that centipede predators switch their diet from feeding on secondary decomposers in rainforest to less ^13^C enriched prey in oil palm plantations. Investigating variations in stable isotope ratios in oribatid mites from temperate ecosystems Gan et al. [52] found oribatid mite species numbers to decline in global change scenarios since trophic specialists will likely go extinct. However, these findings may have been biased as the stable isotope data they used were based on pooled individuals which reduced intraspecific variability. Measuring pooled individuals may reduce the variation in the data and thereby erroneously point to specialist feeding. These restrictions may be circumvented as recent improvements allow to measure stable isotope ratios of small samples [53] including single individuals of soil microarthropod species.

The current study formed part of the interdisciplinary project “Ecological and socioeconomic functions of tropical lowland rainforest transformation systems” (EFForTS), established in Jambi Province, southwest Sumatra (Indonesia) [12]. By measuring natural variations in ^15^N/^14^N and ^13^C/^12^C ratios of individual specimens, we analyzed trophic niches of six soil living oribatid mite species occurring in rainforest and three major rainforest-transformation systems in Southeast Asia, i.e. rubber agroforest (“jungle rubber”), and rubber and oil palm monoculture plantations. We will further refer to those four system in the following as the four land-use systems (rain forest, jungle rubber, rubber, oil palm). We hypothesized that (1) oribatid mite species adapt to environmental changes in transformed ecosystems by shifting their trophic niche, and that (2) the shifts are more pronounced in ^13^C than in ^15^N as changes in land-use systems more strongly affect basal resources (as indicated by ^13^C) than trophic levels (as indicated by ^15^N).

## Material and methods

### Study sites

Soil samples were taken in two regions of Jambi Province, Bukit Duabelas (2° 0’ 57” S, 120° 45’ 12” E) and Harapan (1° 55’ 40” S, 103° 15’ 33” E). In each region four different land-use systems were investigated: rainforest, jungle rubber, rubber and oil palm plantations [12]. Rainforest sites were secondary rainforest which had been selectively logged about 20–30 years ago. Jungle rubber originated from enrichment of rainforest with rubber trees (*Hevea brasiliensis*) and includes rainforest trees. Jungle rubber sites were used to represent rainforest conversion systems of low land-use intensity lacking fertilizer input and herbicide application. Rubber as well as oil palm (*Elaeis guineensis*) monocultures were intensively managed plantations of an average age of 13 to 14 years. These systems were chosen to represent high land-use intensity plantation systems. Four replicates of each land-use system (rainforest, jungle rubber, rubber and oil palm plantations) in the two landscapes (Bukit Duabelas, Harapan) were established, resulting in 32 plots; in each plot samples were taken from three subplots, resulting in a total of 96 samples. Each plot spanned 50 x 50 m and the subplots 5 x 5 m [12]. For more details of the study site see Drescher et al. [12]. At both landscapes (Bukit Duabelas, Harapan) acrisols dominated. Soils with a clay texture dominanted in Bukit Duabelas, whereas soils with a sandy loam texture dominated in Harapan. All study sites were at similar altitudes varying between 50 and 100 m a.s.l. [54].

### Sampling, extraction and species determination

Samples of 16 x 16 cm comprising the litter layer and the underlying 0–5 cm of the mineral soil were taken in October/November 2013. The two layers were separated, transported to the laboratory and extracted by heat [55]. Oribatid mites were determined to species / morphospecies level using Balogh & Balogh [56] and ascribed to feeding guilds including lichen feeders, primary decomposer, secondary decomposer/fungal feeders and predators/scavengers based on Maraun et al. [38]. Species and morphospecies were documented by taking pictures, linked with morphological traits and species identification numbers (species ID), and included into Ecotaxonomy database (http://ecotaxonomy.org/). Animals were stored in 70% ethanol until further analysis.

### Stable isotope analysis

The six most abundant oribatid mite species of 220 species overall (D. Sandmann, unpubl. data) occurring in each of the land-use systems in both landscapes were selected for stable isotope analysis, i.e. *Plonaphacarus kugohi* (Aoki, 1959) (Ecotaxonomy species ID 405729), *Protoribates paracapucinus* (Mahunka, 1988) (Ecotaxonomy species ID 405671), *Scheloribates praeincisus* (Berlese, 1910) (Ecotaxonomy species ID 405449), *Bischeloribates mahunkai* Subías, 2010 (Ecotaxonomy species ID 405450), *Rostrozetes* cf. *shibai* (Aoki, 1976) (Ecotaxonomy species ID 405389), und *Rostrozetes* sp. 1 (Ecotaxonomy species ID 405478). In total, 100 individuals of *S*. *praeincisus*, 75 of *R*. cf. *shibai*, 54 of *P*. *paracapucinus*, 44 of *B*. *mahunkai*, 19 of *P*. *kugohi* and 13 of *Rostrozetes* sp. 1 were analyzed.

For calibration of oribatid mite stable isotope values we measured stable isotope values of leaf litter taken from the dried litter material after extraction of the animals (ca. 2.5 g per sample). Prior to stable isotope analysis the litter was dried at 60°C for 24 h and ground in a ball mill (Retsch Mixer Mill MM200, Haan, Germany). For measuring stable isotope values of Oribatida, single individuals were used. Oribatid mite specimens were dried at 60°C for 24 h and weighed into tin capsules. Between one and three individuals from each of the transformation systems of both landscapes were measured (S1 Table). Stable isotope values were determined by a coupled system of an elemental analyzer (NA 1500, Carlo Erba, Milan, Italy) and a mass spectrometer (MAT 251, Finnigan, Bremen, Germany) adopted for the analysis of small sample sizes [53]. The content of ^13^C and ^15^N was expressed using the δ notation with δX (‰) = (R_sample_−R_standard_) / R_standard_ x 1000, with X representing the target isotope (^15^N or ^13^C) and R_sample_ and R_standard_ the ^13^C/^12^C and ^15^N/^14^N ratios, respectively. As standard for ^13^C and ^15^N analyses Vienna PD Belemnite [57] and nitrogen in atmospheric air were used, respectively. Acetanilid was used as internal standard.

### Statistical analysis

Means of δ^13^C and δ^15^N values of the three litter samples per plot were used as plot-specific litter δ^13^C and δ^15^N values. Differences between plot-specific litter δ^13^C and δ^15^N values and the overall mean litter δ^13^C and δ^15^N values (across all plots, landscapes and land-use systems) were used to adjust individual δ^13^C and δ^15^N values of oribatid mites per plot which allowed direct comparison of stable isotope values of oribatid mites across plots. The procedure resembles the calculation of Δ values but allows to present data relative to the overall mean litter δ^13^C and δ^15^N values. Calibrated data were used for all further analysis. Based on these values average δ^13^C and δ^15^N values of oribatid mite species across plots, land-use systems and landscapes were calculated. Further variations in δ^13^C and δ^15^N values within species across the four different land-use systems were inspected using the standard deviation (SD) of stable isotope values within species per plot (S2 Table). Oribatid mites were ascribed to trophic levels assuming a trophic enrichment of ^15^N by 3.4‰ per trophic level except for primary decomposers for which we used a value of 1.7‰ as they typically are less enriched than consumers of higher trophic level [30,58].

Statistical analyses were performed using R v 3.5.2 [59] with R studio interface (RStudio, Inc.). Normality and variance homogeneity were inspected using diagnostic plots. We did not check for overfitting in the model with all species but as we also inspected each species separately and found stable isotope values to vary significantly with land-use systems overfitting in the model with all species is unlikely. Differences in the variation of δ^13^C and δ^15^N values across land-use systems were inspected using a linear mixed effects model as implemented in the *lme4* package [60]. Fixed factors were species identity and land-use system, with ‘PlotID’ included as random factor. Significant differences between fixed factors were inspected using the *Anova* function. Differences in each δ^13^C and δ^15^N values between species were inspected using a linear mixed effects model as implemented in the *nlme* package [61]. Species identity and land-use systems were used as fixed factors and a random factor ‘PlotID’ was included to account for multiple sampling per plot. The significance of the fixed factors were inspected using the *Anova* function. Pairwise differences between the different land-use systems were inspected using the *glht* package [62] with ‘Tukey’s pairwise contrasts’. Data provided in text and figures are given as means ± 1 SD.

## Results

**Fig 1:**
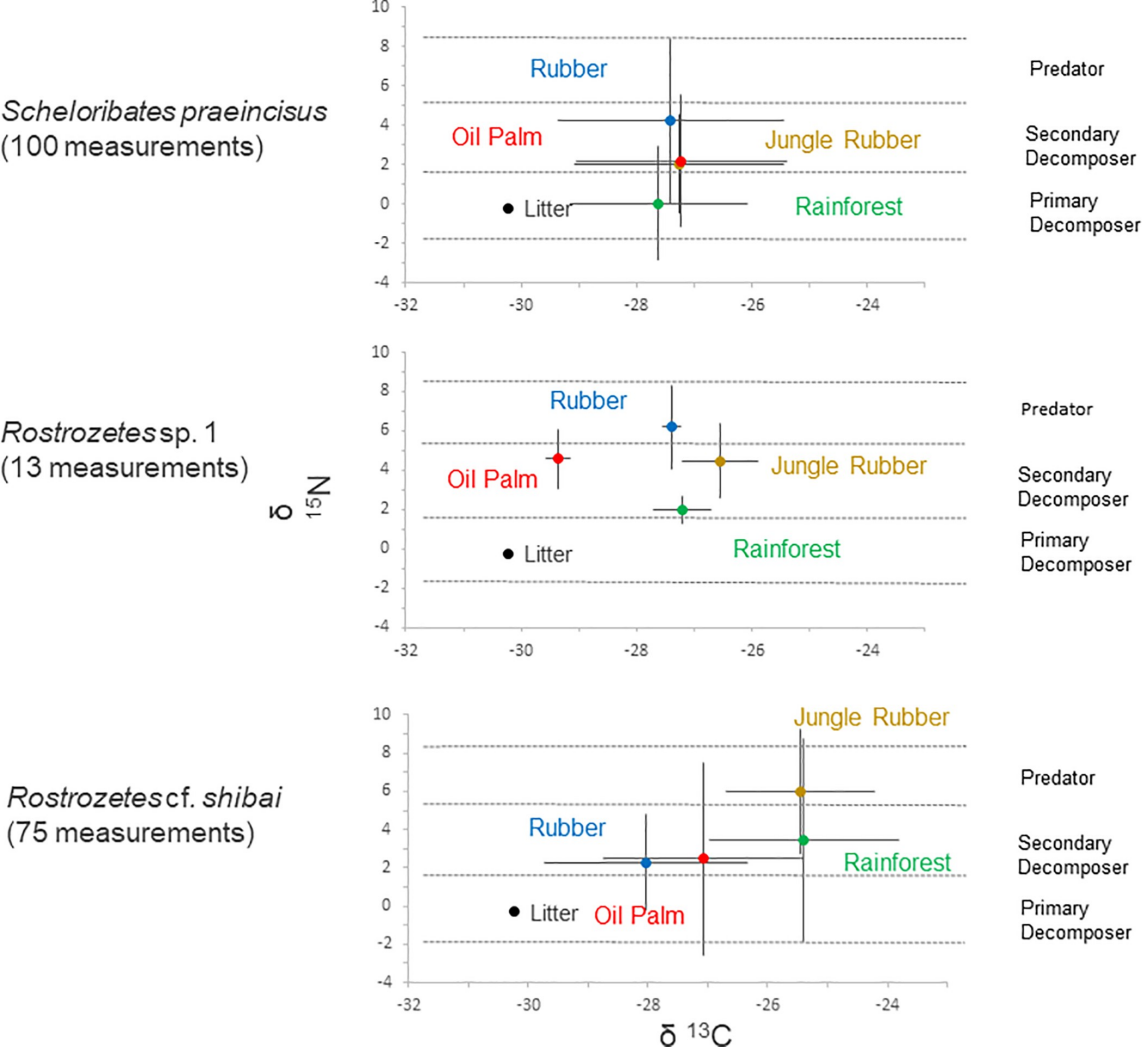
Stable isotope (δ^13^C and δ^15^N) values of oribatid mite species [*Scheloribates praeincisus* (Berlese, 1910), *Rostrozetes* sp. 1 and *Rostrozetes* cf. *shibai* (Aoiki, 1976)] in the four land-use systems studied (rainforest, jungle rubber, rubber and oil palm plantations). Means with standard deviations; numbers of measurements per species are given in brackets. The average stable isotope value of litter used for calibration (see Methods) is given as reference. Dashed horizontal lines reflect boundaries of trophic levels (primary decomposers, secondary decomposers and predators; see Methods). For statistical analysis see text.

Diagnostic plots of standard deviation against mean δ^13^C and δ^15^N showed that the data were distributed normally. Stable isotope values of the combined dataset differed significantly between the six oribatid mite species across land-use systems (χ^2^_5,305_ = 60.56, p < 0.001 for ^13^C, and χ^2^_5,305_ = 78.74, p < 0.0001 for ^15^N). Variation in stable isotope values within species differed significantly between land-use systems for ^15^N but not for ^13^C (χ^2^_3,63_ = 8.53, p = 0.036 and χ^2^_3,63_ = 3.85, p = 0.279, respectively; S2 Fig). Variations in δ^15^N values were similar in rainforest, jungle rubber and rubber plantations (SD of -0.10 ‰, -0.67 ‰ and 0.76 ‰, respectively) but significantly higher in oil palm plantations (SD of 0.62 ‰). Individual mixed effects models for each δ^13^C and δ^15^N values in these species indicated that these shifts were due to changes in δ^15^N values in *S*. *praeincisus* and *R*. cf. *shibai* (χ^2^_3,100_ = 17.14, p < 0.001 for *S*. *praeincisus*, χ^2^_3,54_ = 10.36, p = 0.016 for *R*. cf. *shibai*), with δ^15^N values being lowest in rainforest and highest in rubber plantations in *S*. *praeincisus*, and being highest in jungle rubber and similarly low in rubber and oil palm plantations as well as in rainforest in *R*. cf. *shibai* (Tukey’s HSD test; rubber vs. rainforest p < 0.001 for *S*. *praeincisus*, jungle rubber vs. rainforest p = 0.025, jungle rubber vs. oil palm p = 0.040, jungle rubber vs. rubber p = 0.022 for *R*. cf. *shibai*; Fig 1).

In addition to δ^15^N, shifts in the trophic niche of *R*. cf. *shibai* with land-use system also was due to changes in δ^13^C values and this was also true for *Rostrozetes* sp. 1 (χ^2^_3,13_ = 28.59, p < 0.001 for *Rostrozetes* sp. 1; χ^2^_3,54_ = 13.77, p = 0.003 for *R*. cf. *shibai*). δ^13^C values of *Rostrozetes* sp. 1 in oil palm plantations were significantly lower than those in each of the other land-use systems, whereas δ^13^C values of *R*. cf. *shibai* were significantly lower in oil palm and rubber plantations than in jungle rubber and rainforest (Tukey’s HSD test; oil palm vs. rainforest p = 0.008, oil palm vs. jungle rubber p < 0.001, oil palm vs. rubber p = 0.012 for *Rostrozetes* sp. 1; rubber vs. rainforest p = 0.018, rubber vs. jungle rubber = 0.018 for *R*. cf. *shibai*). Although not significant, δ^15^N values for *Rostrozetes* sp. 1 also varied between land-use systems. Mean ^15^N values classified *Rostrozetes* sp. 1 as secondary decomposer in rainforest, jungle rubber and oil palm plantations, but as predator/scavenger in rubber plantations.

Although stable isotope values of the other three studied oribatid mite species (*B*. *mahunkai*, *P*. *kugohi* and *P*. *paracapucinus*) did not differ significantly among the four land-use systems (Anova; p > 0.05 for all three species), their position varied in isotope space in particular along the δ^13^C axis, resulting in a separation of rainforest and jungle rubber from rubber and oil palm plantations in each of the species thereby resembling the shift in *Rostrozetes* sp. 1 and *R*. cf. *shibai* (S1 Fig).

## Discussion

Based on stable isotope analysis trophic niches of oribatid mites–and soil arthropods in general–have been assumed to vary little at the landscape level [17,24,26,36,63–66] as well as between forest types [26,36]. The results of our study are in contrast to these earlier studies where oribatid mite trophic niches were proposed to be rather stable and narrow.

### Trophic niches of species

The six studied oribatid mite species which occurred in each of the land-use systems spanned three trophic levels including primary and secondary decomposers as well as predators/scavengers, which is conform to earlier studies [24,26,38]. Additionally, intraspecific variation in δ^15^N values were significantly higher in oil palm plantations than in the other three land-use systems. Presumably, this was due to the lack of primary decomposers in oil palm plantations (which only feed on one trophic level, plant litter) and the presence of only higher trophic level species such as secondary decomposers and predators/scavengers, which are more likely to engage in omnivory and intraguild predation. *Bischeloribates mahunkai* grouped as predator/scavenger in rainforest, rubber and oil palm plantations, but as secondary decomposer in jungle rubber. *Protoribates paracapucinus* grouped as secondary decomposer in rainforest and jungle rubber, but as predator in rubber and oil palm plantations. *Scheloribates praeincisus* and *P*. *kugohi* uniformly grouped as primary decomposers in rainforest and as secondary decomposers in the other three land-use systems. Although predominantly grouped as secondary decomposers, *Rostrozetes* sp. 1 and *R*. cf. *shibai* were grouped as predators/scavengers in rubber plantations and jungle rubber. Overall, the results confirm that oribatid mites predominantly function as secondary decomposers feeding on microorganisms, in particular fungi, however, they also indicate that in part they feed on animal prey, presumably nematodes [67,68], or live as scavengers. High trophic position in *B*. *mahunkai* is conform to the suggestion of Rockett [69] that many species of Scheloribatida live as predators. However, lower trophic position of *S*. *praeincisus* suggests that this does not apply uniformly to Scheloribatida as indicated previously [70]. Grouping of *Rostrozetes* sp. 1 and *R*. cf. *shibai* as secondary decomposers (and in part as predators) was unexpected since another species of *Rostrozetes*, *R*. *ovulum*, was shown to live as primary decomposer in a tropical montane rainforest in Ecuador [24]. *Plonaphacarus kugohi* had the lowest ^15^N values and in part was grouped as primary decomposer indicating that this species feeds on litter and microorganisms confirming that Phthiracaridae/Euphthiracaridae often function as primary decomposers [38]. Primary decomposers are characterized by low fractionation of ^15^N which likely is related to “protein sparing”, i.e. the retaining of assimilated N in body tissue rather than excreting it due to low nitrogen supply in litter [66,71–73]. However, recent laboratory studies question that this uniformly applies to oribatid mites [74]. Furthermore, high δ^13^C values of *P*. *kugohi* indicate that this species incorporates calcium carbonate in their exoskeleton [75,76].

### Shifts in trophic niches with land use

Conform to our hypotheses, the studied oribatid mite species shifted their trophic niche with transformation of rainforest into plantation systems, however, this was only significant in three (*S*. *praeincisus*, *R*. cf. *shibai* and *Rostrozetes* sp. 1) of the six studied species, but in trend it also applied to the other three species. This indicates that the ability of the studied oribatid mite species to colonize very different ecosystems at least in part is due to the fact that they are trophically plastic and adapt to the changed environmental conditions in converted ecosystems by shifting their trophic niche. δ^15^N values of *S*. *praeincisus* and *R*. cf. *shibai* differed between the four land-use systems, e.g. δ^15^N values of *S*. *praeincisus* in rubber plantations were almost 4 ‰ higher than in rainforest, whereas δ^15^N values of *R*. cf. *shibai* in jungle rubber were almost 4 ‰ higher than in the other three land-use systems. This indicates that *S*. *praeincisus* as well as *R*. cf. *shibai* alter their resource use with conversion of rainforest/jungle rubber into plantations by shifting its trophic position. *S*. *praeincisus* altered its trophic position from primary decomposer in rainforest to secondary decomposer in plantations, presumably feeding almost exclusively on fungi in the latter. *R*. cf. *shibai* shifted its trophic position from secondary decomposer in rubber, oil palm and rainforest to predator/scavenger in jungle rubber. Notably, *S*. *praeincisus* and *P*. *kugohi* were the only species classified as primary decomposers and they only functioned as primary decomposers in rainforest. This is consistent with earlier studies stressing the lack or scarcity of primary decomposers among oribatid mite species in tropical forest ecosystems [24]. The scarcity of primary decomposers likely is related to the poor litter quality in rainforest ecosystems [77–79], and the results of this study indicates that this is aggravated by conversion of rainforest into plantations as none of the species studied was classified as primary decomposer in plantations. This suggests that the conversion of rainforest into plantation systems aggravates the shortage and poor quality of litter resources for the decomposer community [13,80].

*Rostrozetes* sp. 1 as well as *R*. cf. *shibai* responded in a similar way to the conversion of rainforest into plantation systems as indicated by the shift in δ^13^C values, i.e. changes in the basal resources they are using. In both species δ^13^C values were similar in rainforest and jungle rubber and different from that in oil palm (*Rostrozetes* sp. 1) and oil palm and rubber plantations (*R*. cf. *shibai*). Soil animals typically are enriched by 3–4 δ units in ^13^C as compared to litter due to the “detrital shift” [30,66], and this also was true in the species studied. In *Rostrozetes* sp. 1 and *R*. cf. *shibai* this detrital shift was most pronounced in rainforest and jungle rubber. The more pronounced detrital shift in rainforest and jungle rubber likely reflects a shift in the use of plant litter carbon compounds towards compounds which are easy to access, such as sugars, proteins and (hemi)cellulose, rather than compounds which are difficult to access and have lower δ^13^C values such as lignin [30,66,81–83].

Although stable isotope values in the other three studied oribatid mite species (*B*. *mahunkai*, *P*. *kugohi* and *P*. *paracapucinus*) also varied, these variations were not significant suggesting that their shifts in trophic niches were less pronounced. Notably, in particular the trophic position of *B*. *mahunkai*, classified predominantly as predator, varied little between land-use systems suggesting that this species is unable to switch from animal prey (or carcasses) to feeding on litter or microorganisms. Conform to the significant changes in trophic niches in *S*. *praeincisus*, *Rostrozetes* sp. 1 and *R*. cf. *shibai*, the trophic niches of *B*. *mahunkai*, *P*. *kugohi* and *P*. *paracapucinus* were more similar in rainforest and jungle rubber and separate from those in oil palm and rubber. Also, conform to the former three species, the detrital shift in δ^13^C in *B*. *mahunkai*, *P*. *kugohi* and *P*. *paracapucinus* was less pronounced in rubber and oil palm plantations suggesting that detritivores in these systems benefit from high quality litter of the herb layer (see above). Other studies of oribatid mite families and superfamilies showed results similar to our study on species-level [44]. However, although changes in land use on the trophic structure of soil animals may also be detected at courser taxonomic lever than species, our results indicate that land-use change even affects trophic variability within species, suggesting that to fully appreciate changes in niche space with changes in land use needs high taxonomic resolution and even the level of individuals within species [84–87].

We assumed the shift in trophic niches to be mainly due to changes in the use of basal resources rather than trophic level. Contrary to this hypothesis, the significant shifts in trophic niches in *S*. *praeincisus* and *Rostrozetes* sp. 1 and *R*. cf. *shibai* were due to both changes in the use of basal resources (*Rostrozetes* sp. 1 and *R*. cf. *shibai*) as well as changes in trophic position (*S*. *praeincisus* and *R*. cf. *shibai*). Notably, the shift in δ^15^N values in both of the latter species occurred towards higher trophic positions suggesting that they switched towards including prey of higher trophic levels in converted ecosystems. Overall, this indicates that in particular in primary and secondary decomposers trophic plasticity plays an important role for their ability to colonize a wide range of habitats.

## Conclusions

Of the six species studied occurring across the four land-use systems we detected significant shifts in trophic niches in three of them, but trophic niches of the other three species also varied in a similar way. Notably, the shifts were due to both changes in trophic position (δ^15^N values) as well as changes in the use of basal resources (δ^13^C values) with the shift in trophic position towards higher trophic levels in transformed ecosystems. The observed shifts in trophic niches are conform to the view that oribatid mites are generalist feeders able to change their diet according to changes in resource availability. Notably, the shifts in trophic niches were most pronounced between more natural systems (rainforest and jungle rubber) and high intensity land-use systems (rubber and oil palm plantations). Overall, the results suggest that the ability of oribatid mite species to colonize a wide range of land-use systems including rainforest and monoculture plantations is likely based on trophic plasticity and the ability to shift both their trophic level and the basal resource they rely on.

## Supporting information

S1 Fig Stable isotope (δ^13^C and δ^15^N) values of oribatid mite species [*Bischeloribates mahunkai* Subías, 2010, *Plonaphacarus kugohi* (Aoki, 1959) and *Protoribates paracapucinus* (Mahunka, 1988)] in the four land-use systems studied (rainforest, jungle rubber, rubber and oil palm plantations). Means with standard deviation; numbers of measurements per species are given in brackets. The average stable isotope value of litter used for calibration (see Methods) is given as reference. Dashed horizontal lines reflect boundaries of trophic levels (primary decomposers, secondary decomposers and predators; see Methods).
(TIF)

S2 Fig Plotwise standard deviation of mean stable isotope (δ^13^C and δ^15^N) values of oribatid mite species in the four land-use systems studied (rainforest, jungle rubber, rubber and oil palm plantations) plotted against their stable isotope values (δ^13^C and δ^15^N). For details see S2 Table.
(TIF)

S1 Table Absolute and calibrated (see methods) stable isotope values of oribatid mite individuals studied. Species ID in Ecotaxonomy database (http://ecotaxonomy.org/), species name, landscape, land-use system, absolute values of δ^15^N and δ^13^C values, and calibrated δ^15^N and δ^13^C values. Mean δ^15^N and δ^13^C values of litter used for calibration of -0.24 and -30.23 ‰, respectively.
(XLSX)

S2 Table Standard deviation of mean stable isotope (δ^13^C and δ^15^N) values of oribatid mite species in the four land-use systems studied. Species ID in Ecotaxonomy database (http://ecotaxonomy.org/), species name, land-use system, plotID, replicate, standard deviation of δ^13^C and δ^15^N, mean stable isotope values of δ^13^C and δ^15^N.
(XLSX)
