# Supplementary figures and images for: Shift in trophic niches of soil microarthropods with conversion of tropical rainforest into plantations as indicated by stable isotopes (15N, 13C)
Source: PLoS One. 2019 Oct 25;14(10):e0224520. doi: 10.1371/journal.pone.0224520 (PMC6814230; doi:10.1371/journal.pone.0224520)

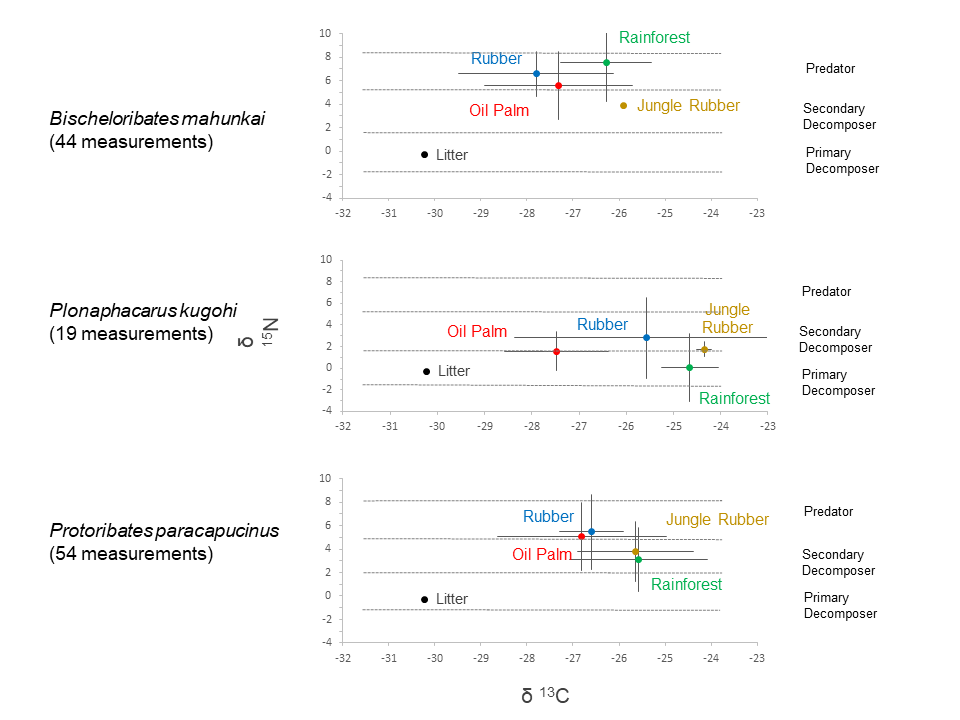

Supplement: S1 Fig — Means with standard deviation; numbers of measurements per species are given in brackets. The average stable isotope value of litter used for calibration (see Methods) is given as reference. Dashed horizontal lines reflect boundaries of trophic levels (primary decomposers, secondary decomposers and predators; see Methods). (TIF) [file pone.0224520.s001.tif]

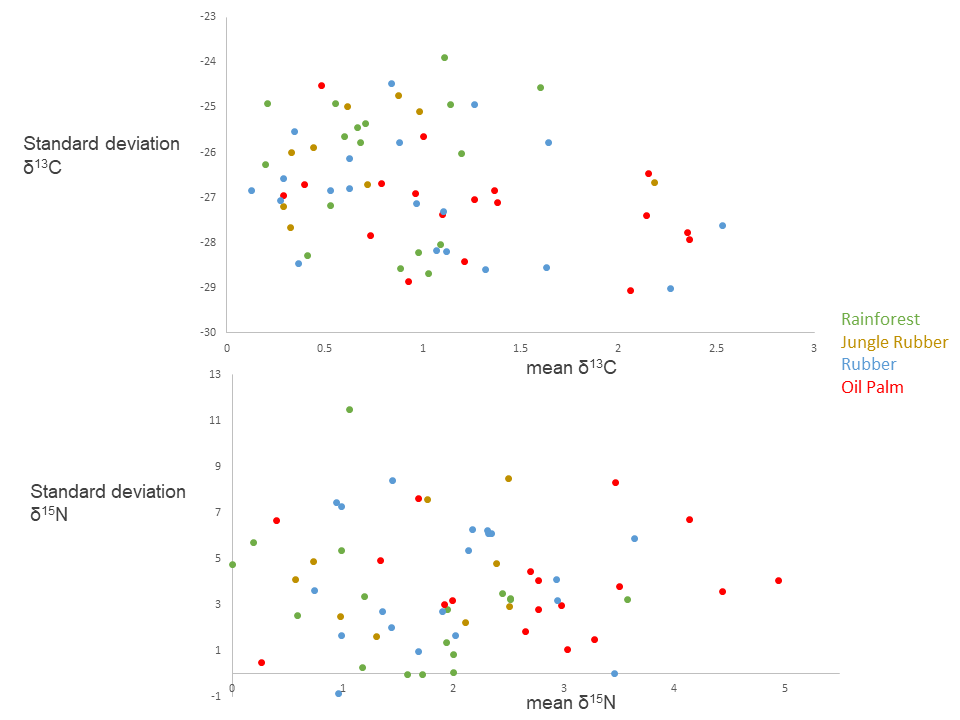

Supplement: S2 Fig — For details see S2 Table. (TIF) [file pone.0224520.s002.TIF]
